# An Adult Arrhythmia in a Child’s Heart: A Case Report of Unexplained Atrial Fibrillation

**DOI:** 10.3390/reports8040264

**Published:** 2025-12-12

**Authors:** Luca Pecoraro, Marta De Musso, Marzia Benelli, Enrico Rosati, Flavia Indrio

**Affiliations:** 1 Pediatric Unit, Ospedale Vito Fazzi, ASL Lecce, 73100 Lecce, Italy; 2 Pediatric Department, University of Bari Aldo Moro, 70121 Bari, Italy; 3 Neonatology and Intensive Care Unit, “Vito Fazzi” Hospital, 73100 Lecce, Italy; 4 Department of Experimental Medicine Pediatric Section, University of Salento Hospital “Vito Fazzi”, 73100 Lecce, Italy

**Keywords:** lone atrial fibrillation, arrhythmia, obesity, childhood obesity, electrocardiogram

## Abstract

**Background and Clinical Significance**: Atrial fibrillation is a rare disorder in the pediatric population in the absence of underlying heart disease. A specific arrhythmia, known as lone pediatric atrial fibrillation, can occur without identifiable structural heart abnormalities. **Case Presentation:** We report a case of a 12-year-old obese child with symptomatic hypertension and atrial fibrillation diagnosed through an electrocardiogram (ECG). **Conclusions:** The patient was rapidly managed with intravenous metoprolol, and he subsequently started cardiologic treatment and clinical follow-up. This case underlines the possibility of performing routine ECGs in the follow-up of obese children.

## 1. Introduction and Clinical Significance

Atrial fibrillation (AF), although rare in children, is rising due to the global epidemic of childhood obesity [1]. On the other hand, it significantly increases the risk of developing isolated pediatric AF in the absence of preexisting heart disease [1]. This pathophysiological link is mediated by complex mechanisms that include epicardial fat accumulation and a chronic state of systemic inflammation and oxidative stress [1,2]. This article presents the clinical case of an obese child who developed symptomatic hypertension and isolated AF, supporting the hypothesis that obesity is a primary and independent risk factor. This clinical case adds another patient to the cases described in other studies [3,4,5]. We therefore highlight the importance of considering the electrocardiogram (ECG) as a routine follow-up tool in obese children for early diagnosis and multidisciplinary management.

## 2. Case Presentation

**Figure 1:**
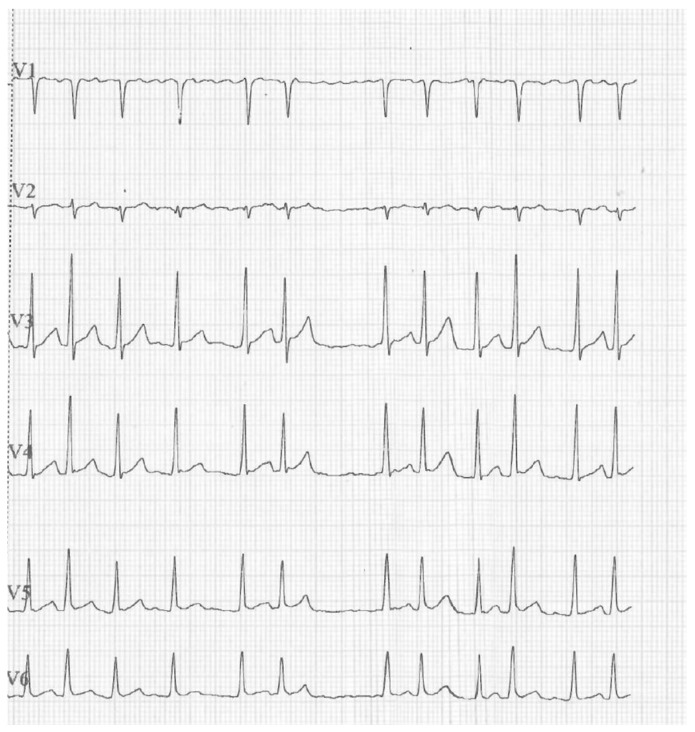
Electrocardiogram: lone atrial fibrillation.

We report an unusual pediatric case report involving a 12-year-old obese child (Body mass index(BMI) 39.3 kg/m^2^) affected by symptomatic hypertension and lone atrial fibrillation. He came to our attention for the first episode of symptomatic hypertension (150/114 mmHg). An electrocardiogram (ECG) was performed, and atrial fibrillation was detected (Figure 1). 

**Figure 2:**
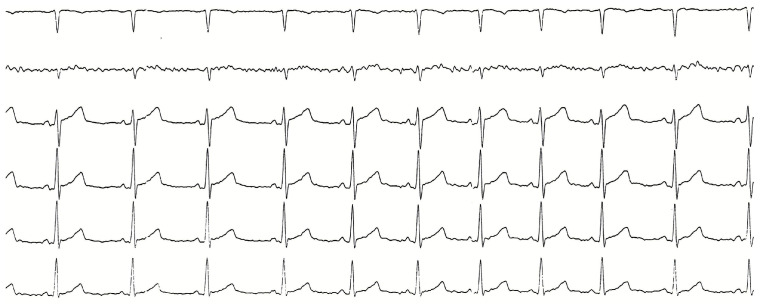
Electrocardiogram performed after restoration of sinus rhythm.

The patient was treated immediately with intravenous metoprolol, and the sinus rhythm was restored in 30 min (Figure 2).

An echocardiography and chest X-ray were performed without any identifiable underlying heart or lung disease. Subsequently, treatment with candesartan and flecainide was started. Considering the normal results of the echocardiogram, no cardiac Magnetic resonance imaging(MRI) has been performed at this time. A 24 h Holter ECG monitoring was performed, confirming a sinus rhythm. Starting from these features, a diagnosis of lone atrial fibrillation was performed. An abdominal ultrasound was performed, with a finding of hepatic steatosis. Lipid and carbohydrate levels were assessed; the lipid profile was normal, while insulin resistance was assessed. The following cardiology tests revealed a normal sinus rhythm and blood pressure. Moreover, the patient underwent a comprehensive diagnostic workup to deepen potential extracardiac causes of the arrhythmic episode, including thyroid function tests, electrolyte assessment, toxicology screening, 24 h urinary catecholamine test, and neurological evaluation. Syndactyly, hyperinsulinism, hypogonadism, and cryptorchidism were identified, and although genetic diagnosis for these manifestations is possible, the patient came to our attention without such a diagnosis. Additional diagnostic workup showed no abnormalities, ruling out secondary causes for the arrhythmia episode. Karyotype analysis showed no abnormalities. Next, treatment was initiated with flecainide (a sodium channel blocker with antiarrhythmic properties) as chronic therapy for its antiarrhythmic efficacy in pediatric atrial fibrillation, candesartan (an angiotensin II receptor antagonist) because it provides stable blood pressure control, metformin, and omega-3 fatty acids. This last compound was prescribed due to the presence of hepatic steatosis. Moreover, he performed a clinical follow-up to lose weight in the Endocrinology Unit. Following hospital discharge, outpatient cardiological follow-up was initiated. A 24 h Holter ECG revealed no abnormalities. Subsequently, a pediatric transesophageal electrophysiological study was performed. During the study, programmed atrial stimulation demonstrated the easy inducibility of atrial fibrillation with a narrow qrs complex. The arrhythmia was sustained, with a mean ventricular rate of 200 beats per minute and required termination via synchronized electrical cardioversion (150 J shock), which successfully restored a sinus rhythm. Based on diagnostic findings and therapeutic response, all previously established pharmacological treatments were maintained, which are the treatments currently in use for this patient. During endocrinological follow-up, the patient experienced weight loss. From a cardiological point of view, the initial treatment was continued, given the reasonable control of blood pressure values and the absence of further arrhythmic episodes. Given the good pharmacological response, radiofrequency ablation was not performed at this time.

## 3. Discussion

### 3.1. Epidemiology and Clinical Context of AF in Children

Atrial fibrillation (AF) is a common arrhythmic condition in adults but infrequent in children [1,5]. It is more prevalent in patients with valvular and congenital heart disease due to increased stress on the left atrium [6]. Recent findings demonstrated a higher risk of atrial fibrillation in obese children and adolescents [7]. Among the causes of atrial fibrillation in children are structural causes (e.g., congenital heart disease, valvular heart disease, cardiomyopathies, etc.), supraventricular tachyarrhythmias, previous cardiac surgery, genetic diseases, and extracardiac causes (e.g., obesity, drugs, obstructive sleep apnoea, thyroid dysfunction, etc.) [7]. According to the Childhood Obesity Surveillance Initiative (COSI), childhood overweight and obesity affect 25% of the population aged 7–9 years. With the increase in the incidence, there is also an increase in obesity-related comorbidities. The COVID-19 pandemic has caused an increase in the prevalence of obesity. Among co-morbidities, cardiovascular effects are the most important [8]. In the pediatric population, the increase in obesity causes a higher incidence of AF [9], even without pre-existing heart disease [9], as in the clinical case described. A specific subtype of atrial fibrillation, known as “Lone Pediatric Atrial Fibrillation” [10], is characterized by a condition of atrial fibrillation without any recognizable underlying heart or lung disease [10]. This condition is also related to obesity [11]. The prevalence is 7.5 per 100,000 [11]. The increase in weight is also associated with increased blood pressure, which can lead to structural changes in the heart, thereby predisposing it to the development of arrhythmia [12]. 

### 3.2. Role of Epicardial Fat and Inflammation in AF Development

Structural and functional changes in the atrial and ventricular myocardium resulting from obesity can lead to abnormalities in atrial and ventricular depolarization and repolarization. There is important evidence about the impact of epicardial fat on the presence, severity and recurrence of AF [13,14,15]. The mechanisms involved are three:

(1) The direct impact of adipocytes from epicardial fat into atrial myocardium, resulting in slow conduction [16,17];
(2) Adipokines secreted by epicardial fat within the pericardial sac could promote paracrine effects on myocardium and consequently fibrosis [18];
(3) Epicardial fat secretes markers of inflammation that result in local pro-inflammatory effects on the adjacent myocardium with increased risk of arrhythmia [19].

Epicardial fat volume is proportional to visceral fat and is strongly linked to atrial remodeling. In a study comparing cardiovascular risk between obese and normal-weight children [20], increased inflammation is one of the most significant mechanisms underlying cardiovascular comorbidity [20]. In addition to left atrial stress, another underlying factor is inflammation of the left atrium caused by the deposition of adipose tissue [9]. The presence of epicardial fat is directly linked to serum levels of inflammatory markers (MCP-1, IL-1, IL-6, soluble IL-6 receptor, and TNF-α) [21]. In patients with AF, higher levels of inflammatory markers have been found [22,23], including serum C-reactive protein (CRP), heat-shock protein (HSP) β1 (commonly referred to as HSP27), interleukin (IL)-6, IL-8, and tumor necrosis factor-α (TNF-α) [22,23]. 

### 3.3. Electrical and Structural Remodeling Mechanisms in Obesity-Related AF

Hypoxia is among the factors that could mediate a link between obesity, inflammation and AF [24]. In patients with AF, the hypoxia-inducible factor (HIF) is upregulated as a result of the transcription of HIF-1α in adipose tissue, particularly in obese patients [24]. Electrical and structural remodeling of atrial tissue is the basis for the maintenance and progression of AF. Regarding electrical remodeling, the main factors are the downregulation of calcium channels, resulting in a reduction in the refractory period [25] and increased potassium efflux, which results in faster repolarization and hyperpolarization of atrial cells [26,27]. Among the factors influencing repolarization, insulin resistance plays an important role and is often associated with obesity [27]. High IL-6 levels are associated with elevated TNF-alpha levels in plasma among obese individuals [28]. TNF is secreted by fat cells and changes insulin signal transmission in skeletal muscle cells, reducing insulin intolerance. Structural remodeling, in which HIF-1a [29] and TNF-α play a key role, leads to a reduction in contractility, prolonging conduction times and contributing to the maintenance of AF [30]. In a mouse model, TNF-α has been shown to induce a change in connexin-40 expression and activate myofibroblasts through transforming growth factor TGF-β [31]. Another effect of TNF-α that may contribute to the onset of AF is the reduction in calcium ion content in the sarcoplasmic reticulum, resulting in leading to a consequent increase in intracellular calcium during diastole [32]. Another important factor in the electrical remodeling of the atrium is IL-6, which plays a key role in early atrial fibrosis by activating the pSTAT3/STAT3 signaling pathway [33]. Its pro-fibrotic effect is also increased by the suppression of T-reg lymphocyte function, leading to increased expression of α-SMA, type I collagen and type III collagen [34]. IL-6 is also implicated in calcium processing dysfunction in cardiac myocytes [35]. Obesity is associated with increased systemic inflammation, and in fact, lean mice have been found to have more CD4+ T cells, which are essential for the suppression of pro-inflammatory macrophages, unlike obese mice, in which CD8+ T cells have been shown to play a role in the recruitment and activation of pro-inflammatory macrophages [36]. Recruitment of M1 macrophages is also promoted by increased transcription of HIF-1α in adipose tissue [37]. The presence of macrophages in adipose tissue is associated with systemic inflammation, and their presence increases in proportion to body weight [38]. 

### 3.4. Oxidative Stress, Endothelial Dysfunction, and Clinical Implications

In obese children, there is an increase in oxidative stress, which is correlated with the development of AF. Specifically, for every 10% increase in reduced glutathione, the risk of developing AF is 30% higher [39]. The production of reactive oxygen species (ROS) is derived from physiological oxygen metabolism [40], and the mitochondria generate them. Although adipose tissue is poor in mitochondria, its function is important for energy production [41]. In obese individuals, ROS production is increased, and consequently, the exposure of adipose tissue to ROS leads to dysfunctional mitochondrial activity, resulting in adipocyte hypertrophy and adipogenesis [41]. Some minimally invasive biomarkers could measure oxidative stress in subadult populations [42]. Among these biomarkers, there are products of Nucleic Acid Oxidation (e.g., 8-hydroxy-2-deoxyguanosine (8-OHdG) [43]), lipid oxidation (e.g., malondialdehyde (MDA)) and protein oxidation; this last group is not clearly a marker of oxidative stress, but it is associated with chronic diseases and ageing [44]. Children with obesity have higher levels of urinary 8-OHdG and circulating MDA, and lower urinary total antioxidant capacity (TAC) [45,46,47]. Oxidative stress is also associated with endothelial status. The OBELIX study evaluated the effect of different antioxidant therapies on endothelium status [48]. Supplementation with substances that have antioxidant and anti-inflammatory properties, such as resveratrol and curcumin, as well as minerals like zinc, selenium, magnesium, folic acid, and vitamin D, may improve endothelial function [49,50,51] and, consequently, reduce cardiovascular risk. Although AF is a rare entity in children in the absence of congenital heart disease, it should be considered in obese children. This condition presents the same electrophysiological features as AF. Specifically, narrow complex “irregularly irregular” and unrecognizable P waves are the main electrocardiogram features of atrial fibrillation. In many cases, fibrillatory waves can be observed. Typically, the ventricular rate ranges from 80 to 180 beats per minute [52]. It is known that these patients must be strictly monitored for a lot of complications, such as high blood pressure. Specifically, it is demonstrated that although systolic and diastolic blood pressure values of obese children are within normal limits, they are higher than those of non-obese individuals in the same age group [53]. Obese children have been reported to have higher heart rates than normal-weight children in the same age group, increasing their mortality risk. Considering the cardiovascular risk factors associated with obesity, weight control is crucial for maintaining a healthy sinus rhythm [54] and can be considered a fundamental key point in therapeutic strategy [54].

## 4. Conclusions

Since this clinical case adds to other cases described in recent studies [3,4,5], it is important to consider the possibility of performing serial ECGs during the follow-up of pediatric patients with obesity. However, the most recent guidelines from the American Academy of Paediatrics (AAP) do not recommend ECG during routine check-ups for obese children, reserving it for cases where there is a specific clinical indication [55]. About this case report, the history of a child with symptomatic hypertension was related to lone atrial fibrillation. Introducing ECG in the context of follow-up of obese children could prevent these situations and detect this type of arrhythmia before becoming symptomatic. Therefore, this case report underlines how obesity can represent an important risk factor associated with the onset of arrhythmias such as AF. Communication between adipose tissue and the heart, identified as a key factor in obesity-related AF, occurs through both the circulation of inflammatory markers and the presence of epicardial adipose tissue, which influences the atrial wall directly. In this case, systemic inflammation and oxidative stress resulting from the accumulation of adipose tissue may have played a key role. During the follow-up period for patients, such as this case report, it would be essential to observe several aspects, including lifestyle changes, diet quality, and the evaluation of antioxidant supplementation. As part of the follow-up for this patient, further genetic testing could be planned, which could reveal some genetic causes of atrial fibrillation that have not yet been identified.

## Data Availability

The original data presented in the study are included in the article, further inquiries can be directed to the corresponding author.

## Abbreviations

The following abbreviations are used in this manuscript:

AF	Atrial Fibrillation
ECG	Electrocardiogram
COSI	Childhood Obesity Surveillance Initiative
WHO	World Health Organization
CRP	C Reactive Protein
IL	Interleukin
TNF	Tumor Necrosis Factor
HSP	Heat-Shock Protein
HIF	Hypoxia-inducible Factor
TGF	Transforming Growth Factor
ROS	Reactive Oxygen Species
TAC	Total Antioxidant Capacity
